# Three-dimensional animation compression optimization based on structured processing and adaptive spatiotemporal segmentation algorithm

**DOI:** 10.1371/journal.pone.0357382

**Published:** 2026-09-08

**Authors:** Xuan Wang, Xinran Yan, Biao Liu, Kecen Liu, Yan Su

**Affiliations:** 1 College of Art, Suzhou University of Science and Technology, Suzhou, China; 2 Institute of Education, University College London, London, England; Communication University of Zhejiang, CHINA

## Abstract

With the rapid development of virtual reality, game engines, and digital twin technologies, the massive size and significant inter-frame temporal redundancy of three-dimensional (3D) animation data have limited its application in cross-platform transmission and real-time rendering. To design a compression algorithm with high compression efficiency, low reconstruction error, and strong adaptability, this paper proposes a 3D animation compression optimization scheme that integrates structured processing and adaptive spatio-temporal segmentation. First, the unordered vertex data is normalized using an improved equal-cluster K-means (eK-means) algorithm. Then, a 3D animation compression algorithm based on adaptive spatio-temporal segmentation (3DACTAS) is constructed to dynamically generate spatio-temporal segmentation blocks. Finally, an optimization algorithm, I3DACTAS, is proposed by combining boundary editing (BE) and matrix reorganization (MR). Experiments show that the I3DACTAS algorithm achieves an average compression ratio of 6.97 on four datasets, a 28.3% improvement over the comparative algorithm AG-3DAC; the average reconstruction error is reduced by 38.7%, and the average structural similarity index reaches 0.934; mobile animation loading time is reduced by more than 72.2%, and the average rendering frame rate is increased by 44.7%. This research solves problems such as inconsistent segmentation boundaries and incomplete redundancy removal in traditional algorithms, providing efficient technical support for real-time interactive 3D animation across platforms and promoting its widespread application on resource-constrained devices.

## 1. Introduction

In recent years, three-dimensional (3D) animation, as a core visual presentation carrier, has been widely applied in fields such as film and television production, interactive entertainment, and virtual simulation [1–3]. However, 3D animation data usually consists of massive continuous frame vertex coordinates and topological relationships, which have problems such as large data volume, significant inter-frame temporal redundancy, and heterogeneous intra-frame spatial features, which seriously restrict its efficient application in cross-platform transmission, real-time rendering, and resource storage on mobile devices [4,5]. For example, on mobile devices or resource-limited platforms, massive 3D animation data can lead to slow loading and rendering stutters, which seriously affect the user experience. 3D animation compression technology, through specific algorithms and techniques, can remove redundant information in 3D animation data, reduce the amount of data as much as possible, and ensure the quality of animation reconstruction, so as to achieve efficient storage, fast transmission, and real-time rendering [6]. Therefore, designing a 3D animation compression algorithm with high compression efficiency, low reconstruction error, and strong adaptability has become a research hotspot and key technical bottleneck in the field of computer graphics [7].

The key contributions of this study are as follows: (1) The eK-means algorithm was proposed to effectively address the disorder and irregularity issues in 3D animation vertex sets, providing a structured data foundation for subsequent compression processes; (2) The 3DACTAS algorithm was designed with a dynamic decision-making mechanism to adapt to the motion characteristics of different animation data, accurately match spatiotemporal dynamics, and enhance compression efficiency; (3) The I3DACTAS algorithm was introduced to resolve boundary inconsistencies and insufficient correlation mining in decomposition matrices, thereby further reducing reconstruction errors and improving compression ratios. This research aims to provide efficient compression technology support for cross-platform real-time interactive 3D animation scenarios, promoting the widespread application of 3D animation on resource-constrained devices.

## 2. Related work

Many scholars have conducted research on the problems in this field. In order to reduce the bandwidth burden of 3D data, X. Wang et al. introduced the Moving Image Experts Group (MAG) format algorithm to optimize the traditional 3D animation compression algorithm in order to obtain better compression effect. Experimental results showed that the algorithm had excellent compression performance and showed that it had certain practical value in the field of architectural animation application [8]. To efficiently compress 3D mesh animation data, G. Luo et al. proposed a dynamic reshaping model based on spectral clustering. This model further improves the compression effect of 3D mesh sequences by processing spatiotemporal data. The results showed that the model could significantly improve the compression effect on various 3D mesh sequences [9]. To improve the compression of topologically stable mesh animation sequences, T. Qin et al. proposed a new framework based on deep learning. This framework realizes the reconstruction of vertex positions by encoding vertex displacement. The results showed that the method could reduce the bit rate by 25% in lossless compression and shorten the encoding time by 95% in lossy compression [10]. To promote the development of graphics streaming technology in cloud gaming applications, M. Pietr et al. proposed a novel real-time compression scheme for 3D animation meshes in client-GPU cloud gaming environments. The results showed that the compression effect of this scheme could significantly reduce the bandwidth required for data transmission while still ensuring the acceptability of visual effects [11]. To compress the size of 3D files, H. Hoang et al. used embedded deformation technology to extract transformation information between consecutive frames, thereby effectively compressing the data volume of 3D human dynamic meshes. The results showed that this method could efficiently transmit key nodes of transformation information [12]. In the face of the inconsistency between 3D facial motion and facial image changes in 2D view in existing facial video compression schemes, Z. Wang et al. proposed a facial video compression method based on 3D key points and 2D motion information. The results showed that the proposed scheme could achieve reliable video communication even under extremely limited bandwidth conditions (e.g., only 2 kbps) [13].

In summary, existing 3D animation compression technologies can be mainly divided into two categories: geometry-driven and data-driven. Geometry-driven compression relies on manually designed geometric features to uncover redundancy, while data-driven compression captures global temporal relationships; however, both suffer from high computational complexity and excessively long compression times. Most algorithms fail to address the core challenges of the disordered vertex set and the dynamic changes in the number of vertices between frames in 3D animation, resulting in insufficient structure and difficulty in adapting to efficient compression models. Furthermore, traditional spatiotemporal segmentation strategies often employ fixed order or fixed granularity partitioning, failing to dynamically match the spatiotemporal dynamic characteristics of animation data, leading to incomplete redundancy removal and inconsistent reconstruction of segmentation block boundaries. Based on this, this research proposes a 3D animation compression optimization scheme that integrates structured processing and adaptive spatiotemporal segmentation. In terms of structured processing, an innovative improved K-means algorithm (eK-means) with equal cluster sizes is proposed to address the disorder and irregularity of vertex sets. In spatiotemporal segmentation, a Three-Dimensional Animation Compression Algorithm Based on Adaptive Spatio-Temporal Segmentation (3DACTAS) is constructed. This algorithm dynamically determines the segmentation strategy based on data characteristics, accurately matching the spatiotemporal dynamics of the animation data. Furthermore, an optimized algorithm, I3DACTAS, is proposed by combining boundary editing (BE) and matrix reorganization (MR) to further address the issues of inconsistent boundaries and insufficient correlation mining of the decomposed matrix.

## 3. Methods and materials

### 3.1. Structured processing of 3d animation data based on eK-means algorithm

**Fig 1:**
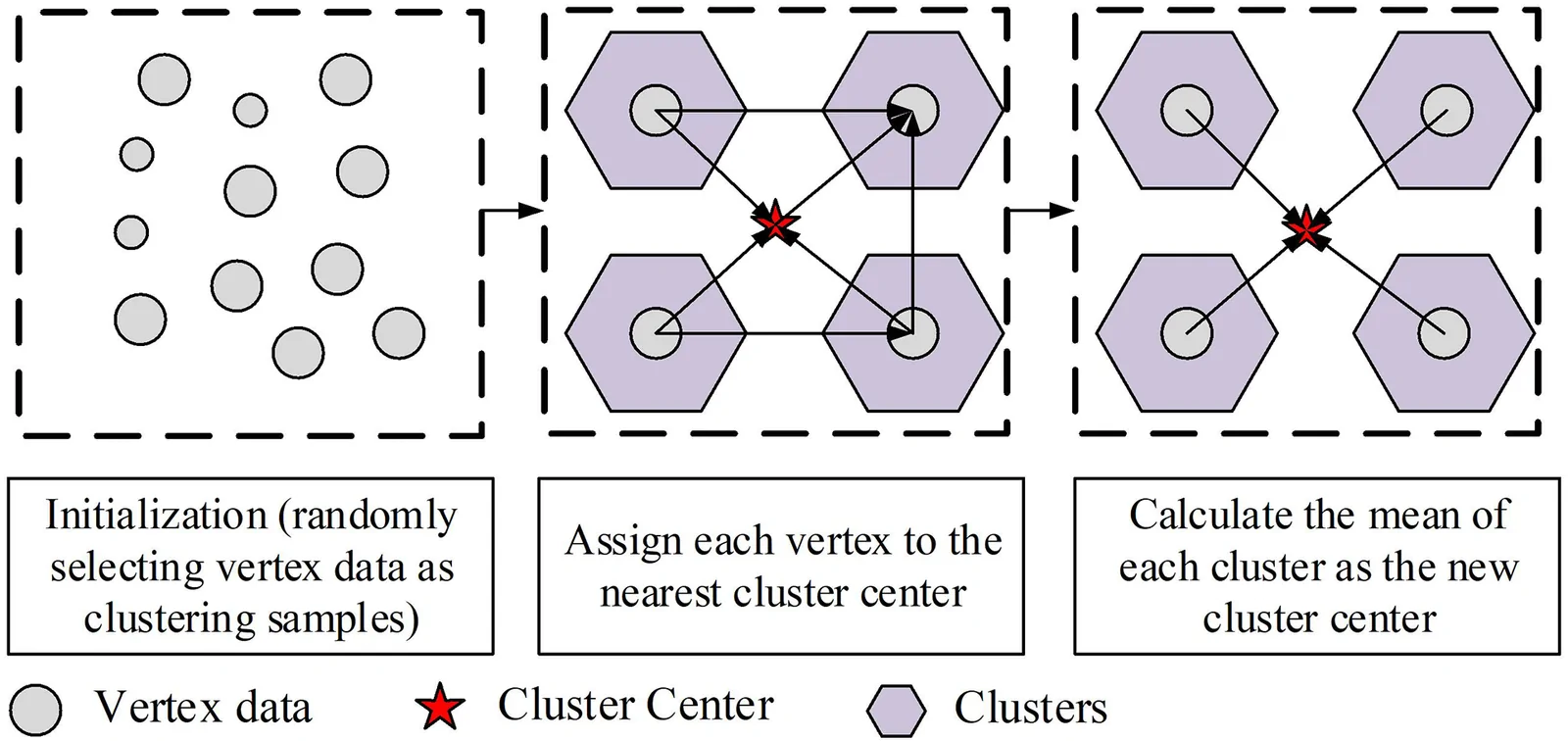
Flowchart of 3D animation data structuring based on eK-means algorithm.

The core component of 3D animation data is the set of vertices in consecutive frames. However, the vertices in 3D animation are arranged randomly within frames, the number of vertices in each frame may change dynamically, and the coordinate data is floating-point. This disorder and irregularity makes it difficult to adapt to compression algorithms [14,15]. When processing such data, the traditional K-means algorithm is difficult to effectively organize vertex data due to the non-fixed cluster size, resulting in insufficient structuring and inability to fully adapt to the subsequent compression process [16]. To this end, the eK-means algorithm was proposed to realize the structuring of 3D animation data. This algorithm ensures that the number of vertices in each cluster does not exceed a specific value by setting equal cluster size, forming a stable equal-sized cluster structure, thus solving the problem of disorder and irregularity of vertex sets. The 3D animation data structuring process based on the eK-means algorithm is shown in Fig 1.

In Fig 1, the process begins with inputting the original vertex set. First, the vertex data from the initial frame is selected as cluster samples, with cluster number computation and centroid initialization performed. Vertices are then assigned to nearest cluster centers using Euclidean distance calculations, forming temporary clusters while strictly controlling cluster size. The clustering process iteratively updates centroids until convergence criteria are met, yielding stable clusters of uniform size. Finally, a vertex index sequence is generated, followed by vertex reordering and coordinate component extraction from the original set to obtain a canonical matrix, completing the structural processing. Concretely, the eK-means algorithm assumes that the vertex set of the 3D animation is $V\in {\mathbb{R}}^{M\times N\times 3}$, where M is the number of animation frames, N is the number of vertices per frame, and the third dimension corresponds to the coordinate (X,Y,Z). To meet the real-time compression requirements, the vertex data of the first frame $V_{\text{1}}$ is selected as the clustering sample. The calculation of the number of clusters K is given by equation (1).


$$\begin{array}{c} K=\left\lceil \frac{N}{64}\right\rceil \end{array}$$ (1)

In equation (1), ⌈•⌉ represents rounding up to ensure that the number of vertices in each cluster does not exceed 64 (i.e., the number of elements in an 8 × 8 sub-block), ultimately forming an equal-sized cluster structure. K vertices are randomly selected from $V_{\text{1}}$ as the initial cluster centers. The Euclidean distance d between each vertex $v_{i}=\left(x_{i},y_{,i}z_{i}\right)$ and each cluster center $\mu_{j}=\left(x_{j},y{,}_{j}z_{j}\right)$ in $V_{\text{1}}$ is calculated, as shown in equation (2).


$$\begin{array}{c} d\left(v_{i},\mu_{j}\right)=\sqrt{(x_{i}-x_{j}{)}^{\text{2}}+(y_{i}-y_{j}{)}^{\text{2}}+(z_{i}-z_{j}{)}^{\text{2}}} \end{array}$$ (2)

Each vertex is assigned to the region corresponding to its nearest cluster center, thus forming K temporary clusters. The number of vertices in each temporary cluster is strictly controlled to ensure that it does not exceed 64. Then, for each temporary cluster, the mean of all vertices in it is calculated, and this mean is determined as the new cluster center, as shown in equation (3).


$$\begin{array}{c} \mu_{j}^{\text{new}}=\frac{\text{1}}{S_{j}}\sum_{v\in C_{j}}v \end{array}$$ (3)

In equation (3), $C_{j}$ represents the jth cluster, and $S_{j}$ represents the number of vertices within the cluster. The cluster assignment and center update steps are repeated until the convergence condition is met: the maximum distance between the cluster centers in two consecutive iterations is less than a preset threshold, or the number of iterations reaches its maximum value. After convergence, K stable clusters are obtained, each cluster corresponding to a set of spatially adjacent vertices. A vertex index sequence Seq is generated based on the clustering results: the vertices within each cluster are sorted in ascending order of cluster center distance, and then the vertex indices of all clusters are concatenated sequentially according to the cluster number. By reordering the vertex dimensions of the original vertex set via Seq, a structured vertex set $V_{\text{new}}\in {\mathbb{R}}^{M\times N\times 3}$ is obtained. Finally, the three coordinate components of $V_{\text{new}}$ are extracted into normalized matrices $V_{\text{x}}$, $V_{y}$, and $V_{z}$, respectively, completing the data structuring process.

### 3.2 Design of a 3D animation compression algorithm based on adaptive spatiotemporal segmentation

**Fig 2:**
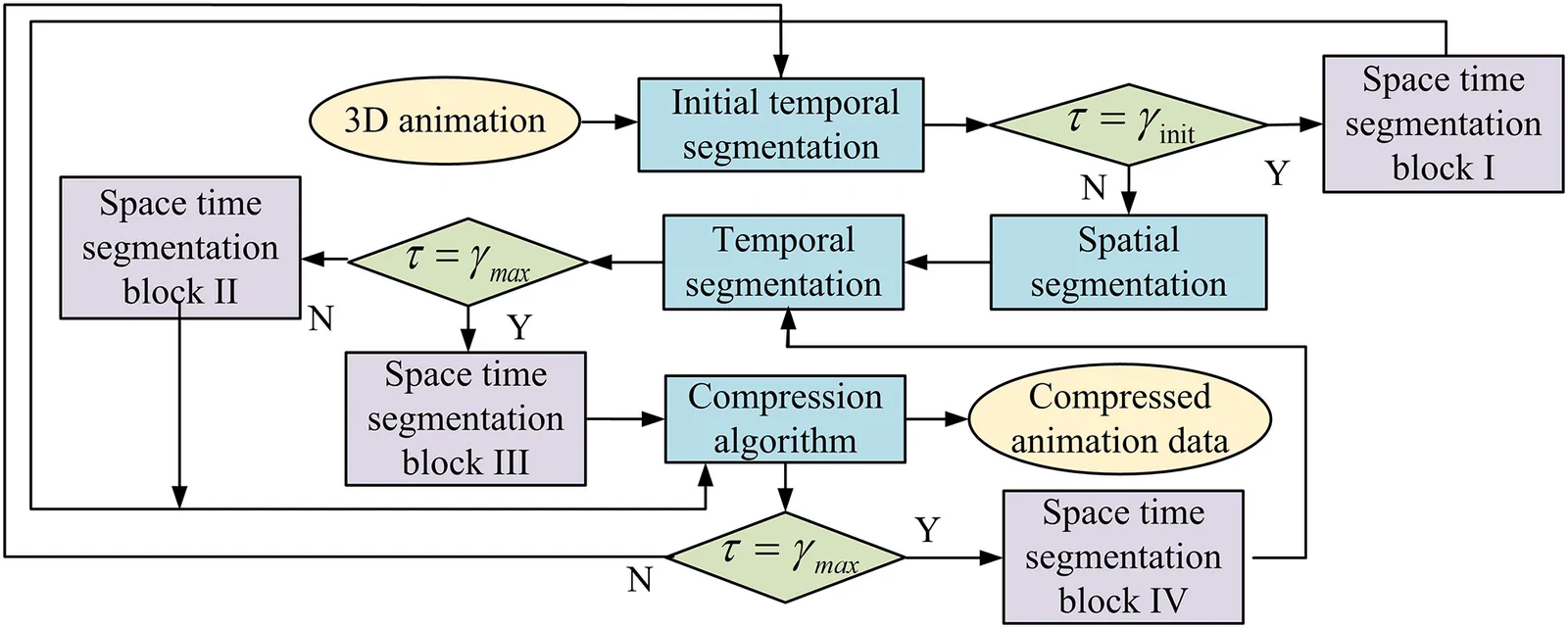
Flowchart of the 3DACTAS algorithm.

After structuring, the 3D animation data has been transformed into a regular coordinate matrix, but there is still significant temporal redundancy between consecutive frames, and the differences in motion characteristics of different regions within a single frame result in insufficient exploitation of spatial redundancy [17,18]. To improve compression efficiency, a 3DACTAS algorithm was designed, and the flowchart of the 3DACTAS algorithm is shown in Fig 2.

Fig 2, the DCATAS algorithm does not require a fixed order of temporal and spatial segmentation. It dynamically determines the segmentation strategy based on data characteristics, decomposing the complete animation sequence into four spatiotemporal segmentation blocks with inherent consistency, thus achieving precise compression. This algorithm retains the regularity advantage of structured data and adapts the spatiotemporal dynamic characteristics of animation data through adaptive segmentation [19,20]. Specifically, based on the structured vertex set $V_{\text{new}}$, the 3D mesh animation to be compressed is defined as shown in equation (4).


$$\begin{array}{c} A=\left(V_{\text{new}}^{f},E\right) \end{array}$$ (4)

**Fig 3:**
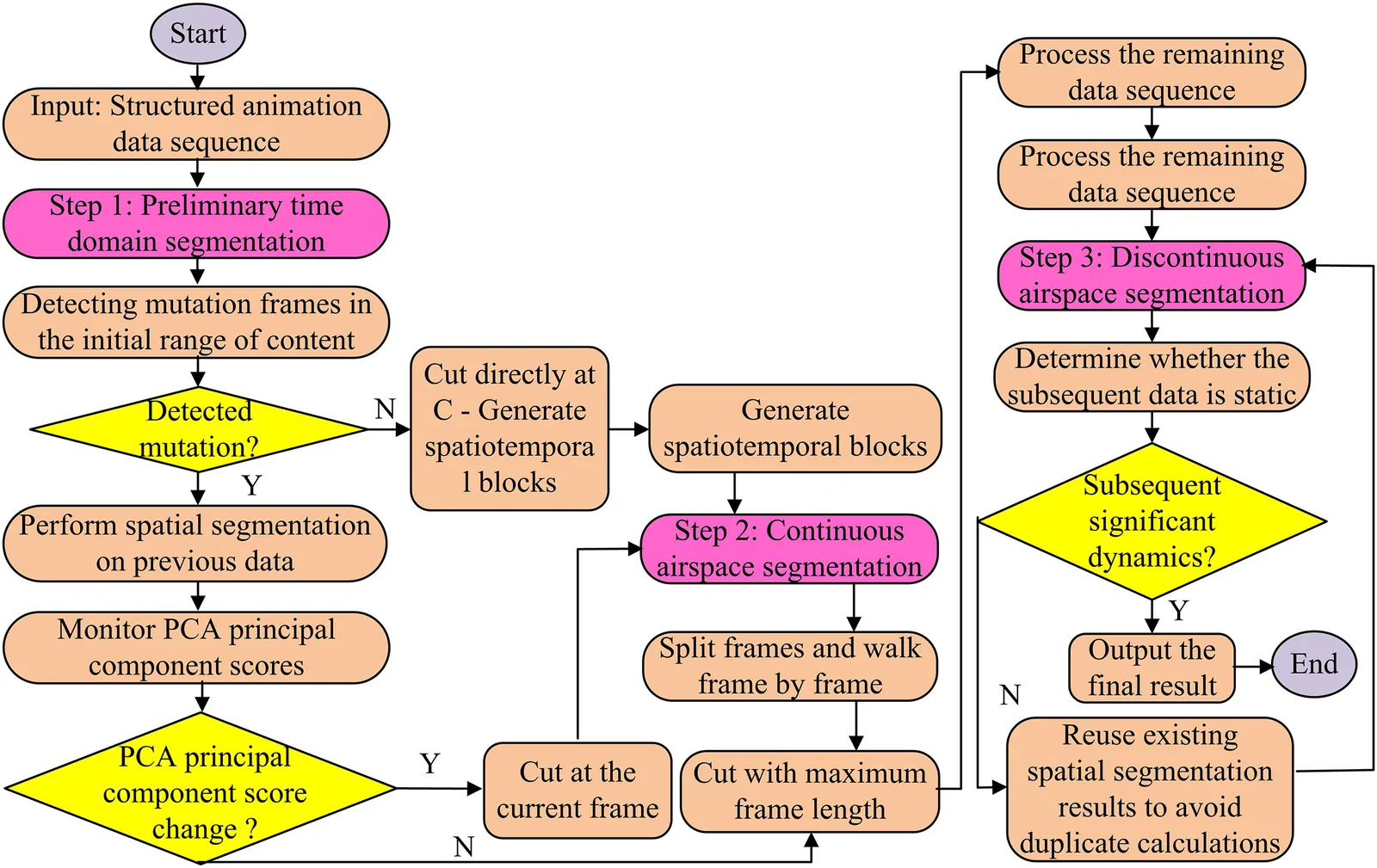
Schematic diagram of adaptive spatiotemporal segmentation steps.

In equation (4), $V_{\text{new}}^{f}$ indicates the fth frame’s structured vertex coordinate matrix, E represents the vertex connection relationship. Next, the adaptive spatiotemporal segmentation stage begins [21,22]. The specific segmentation steps of adaptive spatiotemporal segmentation are shown in Fig 3.

In Fig 3, the first step of adaptive spatiotemporal segmentation is initial temporal segmentation. For the structured animation data, potential segmentation frames τ are detected first within the range of frames $\gamma_{\text{init}}$, with the aim of quickly separating frame sequences with large temporal differences. If no effective segmentation frame is detected (i.e., there is still no significant difference at $\tau =\gamma_{\text{init}}$), a spatiotemporal block is directly generated at $\gamma_{\text{init}}$, without the need for subsequent spatial segmentation. If $\tau ≺\gamma_{\text{init}}$ is detected (abrupt change in inter-frame difference), spatial segmentation is performed on the data of frame length τ first, and then the second step, continuity detection, is performed. The second step is temporal segmentation that continues the spatial segmentation. Within the maximum allowed segmentation frame length $\gamma_{\text{max}}$, the potential segmentation frames τ are moved frame by frame. If the number of principal components changes, the segmentation is performed at the current τ; if the principal components remain unchanged until $\tau =\gamma_{\text{max}}$, the segmentation is performed at the maximum frame length. The third step is temporal segmentation that does not continue the spatial segmentation. For the segmentation blocks generated in the second step, if there are no significant dynamic changes in subsequent data, the existing spatial segmentation results can be reused for direct temporal segmentation, avoiding repeated spatial clustering and reducing computational complexity.

Specifically, for the initial temporal segmentation, the study introduces the kernel embedding distribution and maximum mean difference theory to transform the segmentation problem into a problem of minimizing the correlation between two subsequences [23]. For a potential segmented frame b within frame $\gamma_{\text{init}}$, the correlation between subsequence $\left[V_{\text{new}}^{\text{1}},V_{\text{new}}^{b}\right]$ and $\left[V_{\text{new}}^{b+1},V_{\text{new}}^{\gamma_{\text{init}}}\right]$ needs to be minimized. The objective function is given in equation (5).


$$\begin{array}{c} min_{b\in \left[1,\gamma_{\text{init}}\right]}I\left(\left[V_{\text{new}}^{\text{1}},V_{\text{new}}^{b}\right]\;\;,\left[V_{\text{new}}^{b+1},V_{\text{new}}^{\gamma_{\text{init}}}\right]\right) \end{array}$$ (5)

In equation (5), I represents the correlation between the two subsequences. The similarity of vertex trajectories is measured by the Gaussian kernel function. The correlation calculation and kernel function definition are shown in equation (6).


$$\begin{array}{c} \left\{ \begin{array}{c} @l@{min}_{b\in [1,\gamma_{\text{init}}-w]}-\frac{\text{1}}{\left|T_{\text{1}}\right|\left|T_{\text{2}}\right|}\sum_{i=1}^{\left|T_{\text{1}}\right|}\sum_{j=1}^{\left|T_{\text{2}}\right|}K\left(V_{\text{new},b,i\to i+w},V_{\text{new},b,j\to j+w}\right)+\frac{\text{1}}{|T_{\text{1}}{|}^{\text{2}}}\sum_{i,j=1}^{\left|T_{\text{1}}\right|}K(\cdot )+\frac{\text{1}}{|T_{\text{2}}{|}^{\text{2}}}\sum_{i,j=1}^{\left|T_{\text{2}}\right|}K(\cdot )\\ K\left(V_{\text{new},b,i\to i+w},V_{\text{new},b,j\to j+w}\right)=exp\left(-\lambda |V_{\text{new},b,i\to i+w}-V_{\text{new},b,j\to j+w}{|}^{\text{2}}\right)\; \end{array}\right. \end{array}$$ (6)

In equation (6), $T_{\text{1}}$ and $T_{\text{2}}$ are the vertex trajectory sets of the two subsequences, respectively, and $V_{\text{new},b,i\to i+w}$ represents the vertex trajectory segment near the bth frame with a window size of w. λ represents the kernel function bandwidth parameter. Solving for the minimum correlation degree corresponds to b, which is the initial segmented frame $\tau_{\text{init}}=b$.

The temporal segmentation, which continues the spatial segmentation, is based on the frame blocks generated by the initial temporal segmentation. It divides the region based on the difference in the rigidity of vertex motion. The maximum change in the side length of adjacent vertices within the frame block is calculated. The side length change value is fitted by an exponential distribution. The edge with the smallest change is selected as a rigid edge, and the vertices connected to it are rigid vertices. The rest are non-rigid vertices. Then, a region growing algorithm is used to cluster the rigid vertices to obtain an initial rigid cluster. Finally, the rigid clusters are combined by minimizing the reconstruction error to ensure the intrinsic correlation of the segmented regions. The reconstruction error is shown in equation (7).


$$\begin{array}{c} |\delta_{i}-{\hat{\delta }}_{i}{|}^{\text{2}}=|\delta_{i}-(C[i]+{\hat{\delta }}_{i}){|}^{\text{2}}\; \end{array}$$ (7)

In equation (7), $\delta_{i}$ represents the trajectory vector of the ith vertex, C[i] represents the cluster center vector, and ${\hat{\delta }}_{i}$ represents the trajectory reconstruction vector. Finally, the spatial domain is divided into $N_{g}$ regions, completing the spatiotemporal collaborative segmentation.

For the temporal segmentation of the non-continuous spatial segmentation in the third step, in order to quantitatively judge the degree of dynamic change of the subsequent frame sequence, the inter-frame vertex trajectory similarity threshold θ is introduced to calculate the vertex trajectory similarity S between the current frame and the last frame of the previous segmentation block, as shown in equation (8).


$$\begin{array}{c} S=\frac{\text{1}}{n}\sum_{i=1}^{n}\frac{{\vec{T}}_{c,i}\cdot {\vec{T}}_{l,i}}{\left|{\vec{T}}_{c,i}\right|\cdot \left|{\vec{T}}_{l,i}\right|} \end{array}$$ (8)

In equation (8), ${\vec{T}}_{c,i}$ represents the trajectory vector of the ith vertex in the current frame, ${\vec{T}}_{l,i}$ represents the trajectory vector of the nth vertex in the last frame of the previous segmentation block, and i represents the number of vertices. When S≥θ, it is determined that the dynamic changes of the subsequent frame sequence are not significant, and the spatial domain segmentation result is reused for temporal domain extension; when S<θ, the current extension is terminated, and the initial temporal domain segmentation process is re-executed to ensure that the segmentation block always has good internal feature consistency.

After completing the adaptive spatiotemporal segmentation and generating L spatiotemporal blocks, the compression stage begins. In this stage, the study further adopts a combined strategy of “Principal Component Analysis (PCA) dimensionality reduction + lossless compression.” Specifically, the principal components of the spatiotemporal block are first extracted by PCA decomposition, and the first p principal components with a cumulative contribution rate of not less than α are retained to achieve data dimensionality reduction. PCA decomposition and reconstruction are shown in equation (8).


$$\begin{array}{c} \left\{ \begin{array}{c} @l@\delta_{i}=B_{i}\times C_{i}+A_{i}\\ {\hat{\delta }}_{i}\approx B_{i}(1:p)\times C_{i}(1:p)+A_{i}\; \end{array}\right. \end{array}$$ (9)

In equation (9), $B_{i}$ represents the data projection matrix, $C_{i}$ is the principal component matrix, and $A_{i}$ is the mean vector. The original data is approximated by the first p principal components. Then, the dimension-reduced $B_{i}$, $C_{i}$, and $A_{i}$ are converted into binary data using the Zlib lossless compression algorithm. The final compression result is shown in equation (10).


$$\begin{array}{c} \text{Compress}\left(A\right)=\sum_{l=1}^{L}\psi_{l}\; \end{array}$$ (10)

In equation (10), $\psi_{l}$ represents the lossless compression result of the lth spatiotemporal block, and the compressed data of the complete animation is obtained by accumulation. This adaptive spatiotemporal segmentation algorithm adapts to the motion characteristics of different animation data through a dynamic decision-making mechanism, which not only avoids the insufficient adaptation of fixed segmentation strategies to heterogeneous data, but also maximizes the removal of redundancy through spatiotemporal collaborative segmentation.

### 3.3. Optimization of 3D animation compression algorithm based on BE and MR

3DACTAS algorithm achieves accurate segmentation and preliminary compression of structured data in 3D animation, there are still two areas for optimization: First, when adjacent spatiotemporal segments are independently subjected to PCA dimensionality reduction, the difference in principal component selection can easily lead to inconsistent data reconstruction at block boundaries, destroying the global correlation of structured data; second, the matrix obtained by PCA decomposition is directly converted into a one-dimensional vector for lossless compression, failing to fully explore the intrinsic correlation between the same type of decomposition matrices in different segments, resulting in incomplete redundancy removal [24,25]. To address this issue, this study proposes a joint optimization strategy that integrates BE and MR to optimize and improve the 3DACTAS algorithm (hereinafter referred to as the I 3DACTAS algorithm).

To address the boundary inconsistency problem, this study utilizes the uniformly sized regular vertex clusters generated by the eK-means algorithm and designs a BE method based on structured cluster features. This method first expands the boundary of each structured cluster block by 2-neighborhood. Then, combining the structured vertex index sequence from the 3DACTAS algorithm, it locates the overlapping cluster regions of adjacent segmentation blocks and extracts the mean vertex coordinates as boundary reference features. During decompression and reconstruction, the boundary reference features of the current block and adjacent blocks are weighted and fused to correct the PCA dimensionality reduction error, ensuring a smooth transition of boundary vertex coordinates and avoiding abrupt changes.

**Fig 4:**
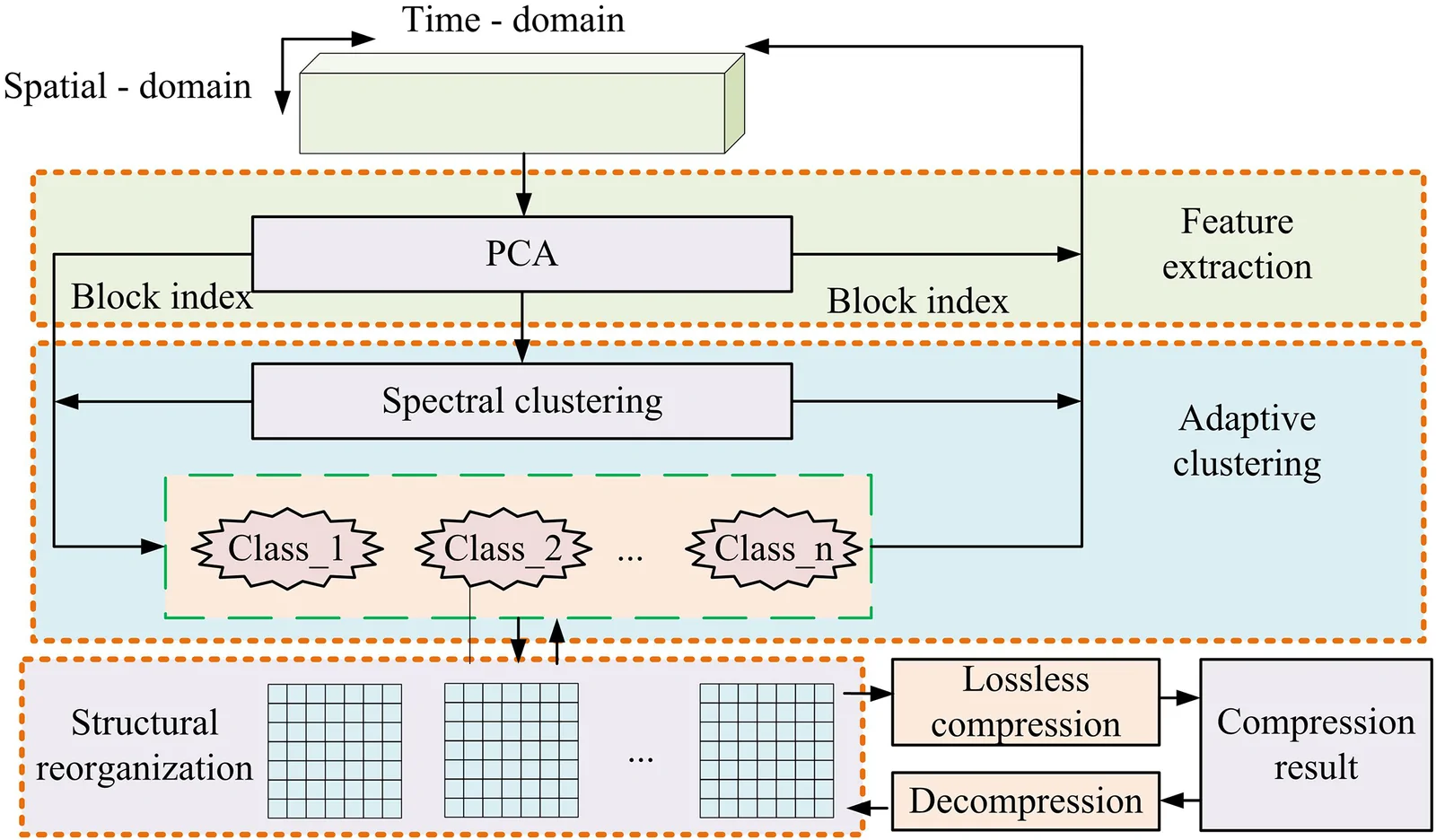
MR optimization flowchart.

To address the issue of untapped potential correlations in the decomposition matrix, this study proposes an MR optimization method based on spectral clustering, the overall process of which is shown in Fig 4.

In Fig 4, MR optimization improves the redundancy removal efficiency of subsequent lossless compression through three core steps: feature extraction, adaptive clustering, and structured recombination. The premise of MR is to accurately measure the similarity of different decomposition matrices. Continuing the PCA decomposition results of the 3DACTAS algorithm, the projection matrix, principal component matrix, and mean vector of all spatiotemporal segments are denoted as a set X. Since the decomposition matrix sizes of different segments differ, directly calculating the Euclidean distance has limitations [26,27]. Therefore, this study uses histograms to characterize the distribution characteristics of matrix data and measures the distribution similarity through Earth Mover’s Distance (EMD), as shown in equation (11).


$$\begin{array}{c} \omega_{i,j}=EMD\left(H\left(X_{i}\right),H\left(X_{j}\right)\right)\; \end{array}$$ (11)

In equation (11), $H\left(X_{i}\right)$ represents the histogram feature of the decomposition matrix $X_{i}$, and $\omega_{i,j}$ represents the similarity measure of the two matrices. The smaller the value, the closer the distributions are. The essence of EMD distance is to solve for the minimum transportation cost between the two distributions, as shown in equation (12).


$$\begin{array}{c} min\sum_{i,j}f_{i,j}d_{i,j}\; \end{array}$$ (12)

**Fig 5:**
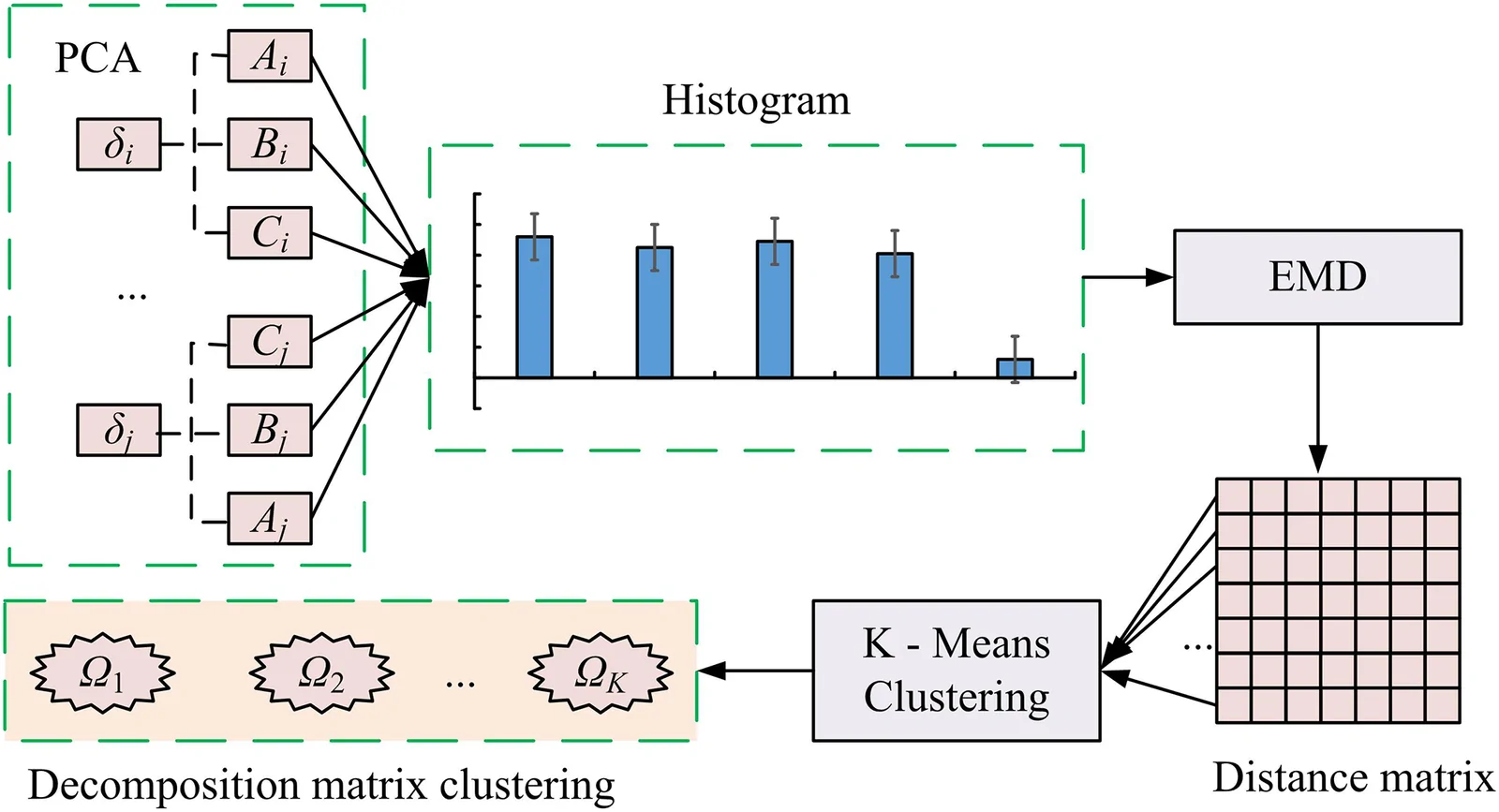
Flowchart of spectral clustering.

In equation (12), $f_{i,j}$ represents the element migration between distributions, and $d_{i,j}$ represents the Euclidean distance between elements. After similarity measurement, spectral clustering algorithm is used to classify the decomposed matrix, and the process is shown in Fig 5.

In Fig 5, spectral clustering, through four steps—constructing a similarity graph, calculating the Laplacian matrix, eigenvalue decomposition, and clustering—can effectively handle the clustering problem of non-convex data and adapt to the complex distribution characteristics of the decomposition matrix. The number of clusters M is determined using an adaptive calculation method, combined with the number of segmentation blocks, as shown in formula (13).


$$\begin{array}{c} K=\left\lceil \sqrt{3M}\right\rceil +1 \end{array}$$ (13)

**Fig 6:**
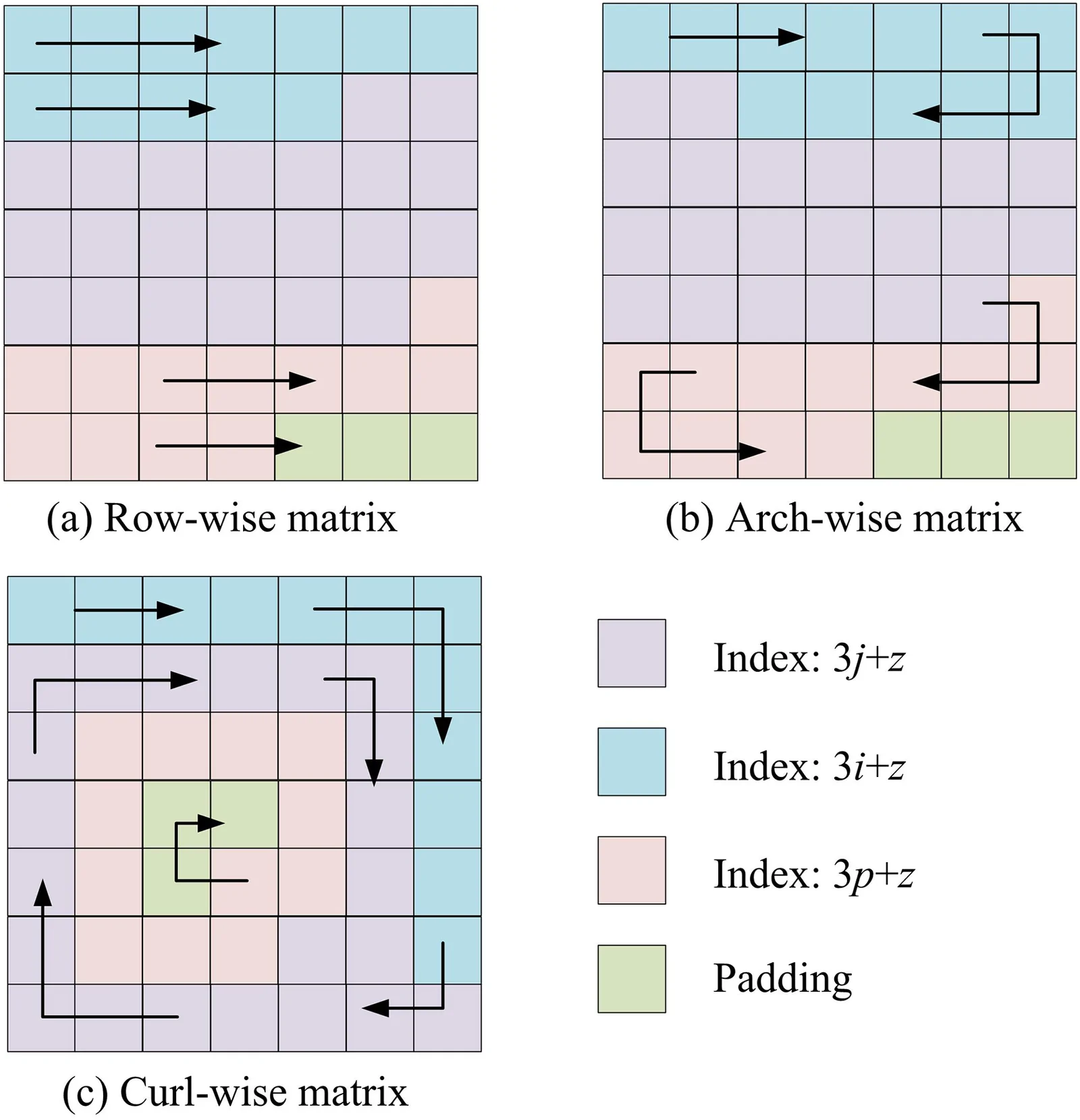
Schematic diagram of MR reconstruction method.

In equation (13), ⌈•⌉ represents the rounding up operation, Mrepresents the number of clusters. After clustering, the decomposition matrices within the same category are restructured. To maximize the “contact area” of similar data and improve the efficiency of lossless compression, three restructuring methods are designed, as shown in Fig 6.

In Fig 6, the row-first filling method starts from the top left corner and fills rows sequentially, continuing after reaching the specified number of columns. The arched filling meth od fills rows to the end of the column and then fills the next row in the opposite direction, forming an alternating arrangement. The spiral filling method starts from the top left corner and fills clockwise in a spiral pattern until a regular square matrix is formed. During the recombination process, gaps caused by differences in matrix sizes are filled with 0. This method, while ensuring the regularity of the recombination, significantly reduces computational complexity and does not affect the compression effect compared to mean filling, maximum filling, and other methods. The recombination dimension is determined by the total number of elements in the same category of matrices, as shown in equation (14).


$$\begin{array}{c} D=\sqrt{\sum_{k=k_{\text{0}}}|X_{k}|} \end{array}$$ (14)

In equation (14), $k_{\text{0}}$ represents the target clustering category, $X_{k}|$ represents the number of elements in the kth decomposition matrix within the category, and D represents the dimension of the recombined matrix.

## 4. Results

### 4.1. Dataset selection

To verify the effectiveness of the proposed 3DACTAS and I3DACTAS algorithms, the study selected four 3D animation datasets—“Cloth,” “Chicken,” “Horse,” and “Michael”—for performance testing. The four datasets are all sourced from the public 3D animation dataset repository. To meet the experimental requirements of the study, targeted construction and preprocessing were performed on the public datasets: ① Cloth dataset: The original data consists of cloth fluttering animations, with a total of 200 frames and 10,240 vertices per frame. During construction, 120 frames with stable motion states and distinct deformation features were selected, and the number of vertices per frame was downsampled to 8,192, retaining the core features of random fluttering of flexible bodies; ② Chicken dataset: The original data consists of chickens pecking animations, with a total of 200 frames and 5,120 vertices per frame. During construction, 150 effective action frames were selected, and the number of vertices per frame was downsampled to 4,096, retaining the coupled features of local deformation at joints and overall rigid motion; ③ Horse dataset: The original data consists of horses running animations, with a total of 250 frames and 8,192 vertices per frame. During construction, 200 consecutive action frames were selected, and the number of vertices per frame was downsampled to 6,144, emphasizing the features of severe joint deformation; ④ Michael dataset: The original data consists of human walking and waving animations, with a total of 250 frames and 6,144 vertices per frame. During construction, 180 consecutive action frames were selected, and the number of vertices per frame was downsampled to 5,120, retaining the features of regular human motion and multi-part collaborative deformation. After preprocessing, the four datasets underwent normalization, mapping the vertex coordinates to the [0,1] interval to eliminate the impact of coordinate scale differences on experimental results. Finally, the experimental datasets required for the study were constructed, covering typical 3D animation types such as flexible, rigid-flexible hybrid, and human motion, with reasonable vertex scale and frame length distributions, enabling comprehensive testing of the algorithm’s generality and robustness. The experimental environment and algorithm parameter settings are shown in Table 1.

**Table 1:** Experimental environment and algorithm parameter settings.

Classification	Specific parameters	Numerical explanation
Experimental hardware environment	CPU	Intel Core i7-12700H
	GPU	NVIDIA GeForce RTX 3060
	EMS memory	32GB DDR5 4800MHz
	Storage	1TB NVMe SSD
Experimental software environment	OS	Windows 11
	Programming language	Python 3.9.16
	Scientific computing library	NumPy 1.24.3, SciPy 1.10.1
	Visualization Tools	Matplotlib 3.7.1
eK-means	Maximum cluster size	64
	Convergence threshold	1e-4
	Maximum iteration count	100
3DACTAS	Cumulative contribution rate of PCA	≥95%
	Maximum segmentation frame length	30
	Gaussian kernel bandwidth parameter	0.1
	Rigid edge determination threshold	0.02
I3DACTAS	Extension of boundary expansion	2
	coefficient of cluster number in cluster analysis	0.3

**Fig 7:**
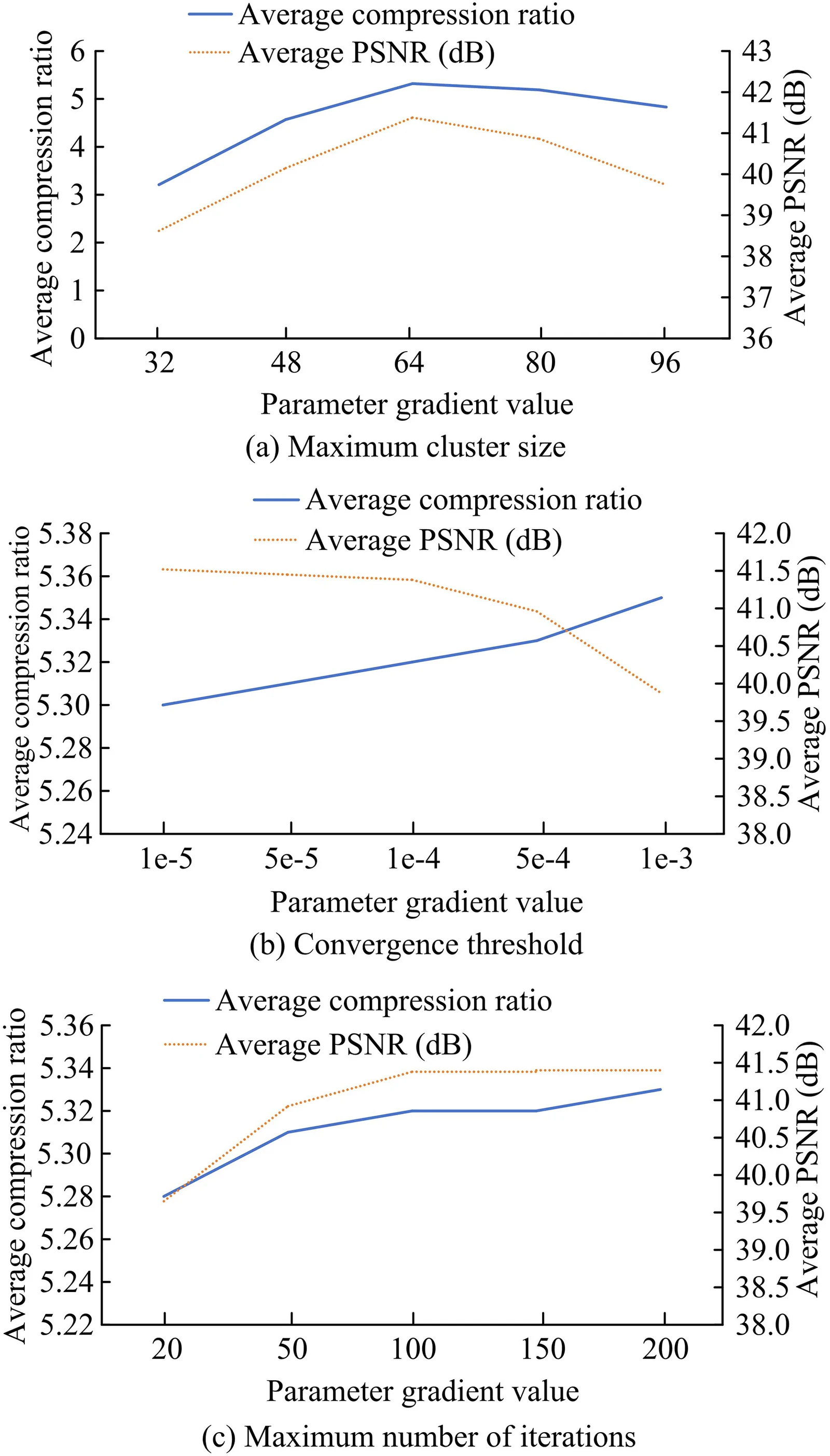
Sensitivity analysis of eK-means algorithm parameters (Evaluation metrics: Compression ratio and PSNR (dB); Experimental hardware: Intel i7-12700H + RTX3060).

First, for the three core parameters of the eK-means algorithm, the compression ratio and peak signal-to-noise ratio (PSNR) were used as evaluation indicators to conduct a sensitivity analysis. The experimental results are shown in Fig 7.

**Fig 8:**
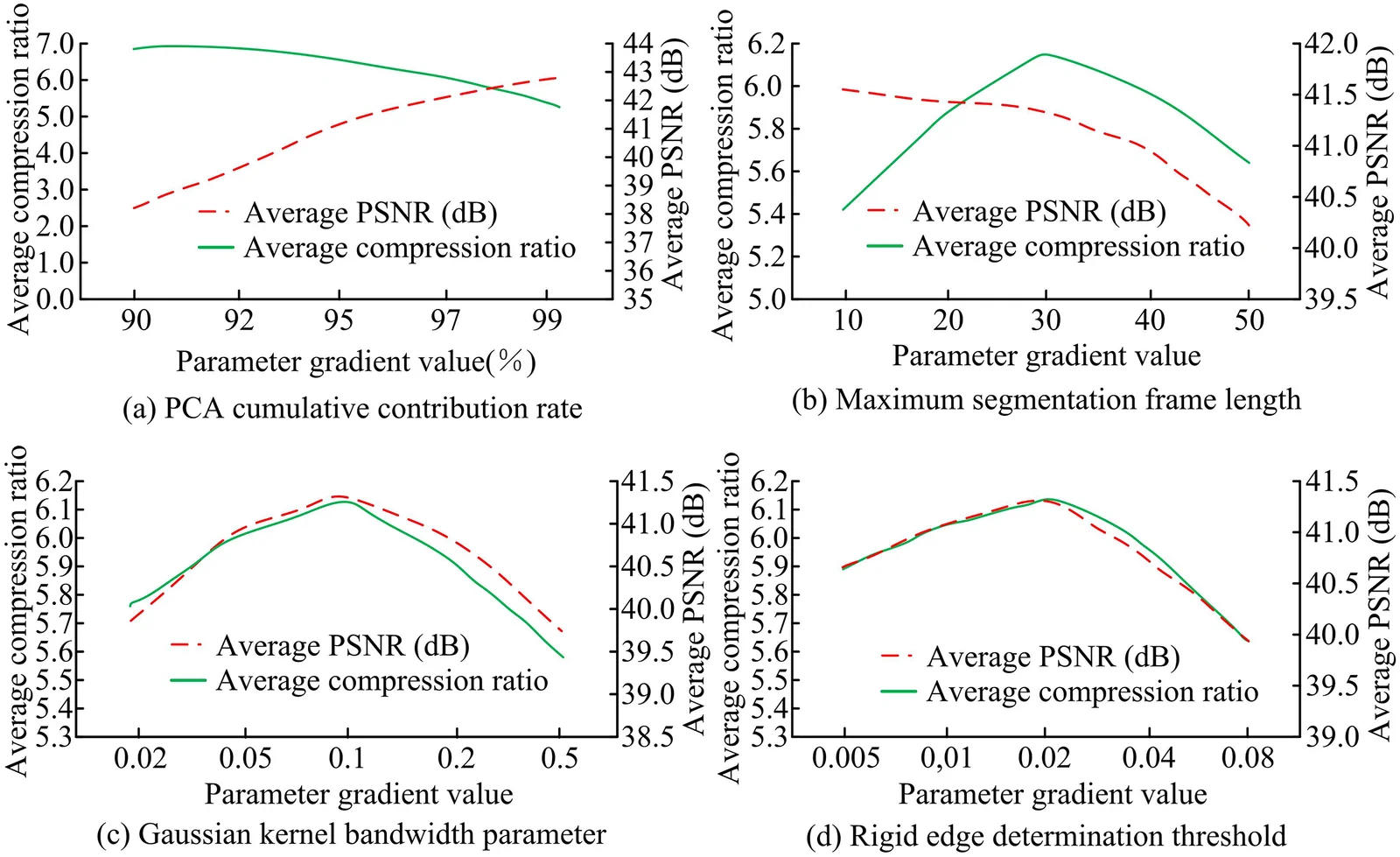
Sensitivity analysis of 3DACTAS algorithm parameters.

As shown in Fig 7(a), the maximum cluster size had a significant impact on the indicators. Within the range of 32–64, both the compression ratio and PSNR increased with increasing cluster size, reaching their optimal values at 64 (compression ratio 5.32, PSNR 41.38 dB). Beyond 64, both indicators declined due to decreased vertex correlation within the cluster. Fig 7 (b) shows that the convergence threshold’s influence was concentrated on PSNR. Within the range of 1e^-5^ to 1e^-4^, PSNR fluctuations were less than 0.6 dB. Beyond the threshold of 5e^-4^, PSNR decreased rapidly, while the compression ratio remained relatively stable (fluctuations ≤ 0.05). Fig 7 (c) shows that the maximum number of iterations significantly improved PSNR within the range of 20–100, but converged after 100 iterations. Further increasing the number of iterations had minimal improvement on either indicator, indicating that 100 iterations were sufficient to meet the clustering stability requirements. In summary, the optimal parameter combination for eK-means was a maximum cluster size of 64, a convergence threshold of 1e^−4^, and a maximum number of iterations of 100. For the four core parameters of the 3DACTAS algorithm, the compression ratio and PSNR were used as evaluation indicators. Sensitivity analysis was carried out based on the structured dataset, and the experimental results are shown in Fig 8.

As shown in Fig 8, the cumulative contribution rate of PCA was negatively correlated with the compression ratio and positively correlated with PSNR. A balance between compression efficiency and reconstruction quality could be achieved at 95%. The compression ratio was optimal when the maximum segmentation frame length was 30. Too short a frame length led to too many segmentation blocks, increasing redundancy, while too long a frame length disrupted inter-frame correlation, causing a decrease in PSNR. The Gaussian kernel bandwidth parameter was optimal for both metrics when it was 0.1. Deviating from this value would reduce the accuracy of inter-frame difference detection and affect the segmentation effect. The rigid edge determination threshold was optimal when it is 0.02. Too small a threshold resulted in overly fine division of rigid regions, while too large a threshold would miss flexible deformation regions, both of which would reduce compression and reconstruction performance. In summary, the optimal parameter combination for 3DACTAS was a cumulative PCA contribution rate of 95%, a maximum segmentation frame length of 30, a Gaussian kernel bandwidth of 0.1, and a rigid edge determination threshold of 0.02.

To evaluate the parameter sensitivity of the I3DACTAS algorithm on unseen datasets, we selected the “Humanoid” dataset (human dance animation, 180 frames, 5120 vertices per frame) from a public dataset repository that was not used for training or optimization. The experiment tested the impact of core hyperparameters on SSIM and validated parameter generalization. Experimental results are presented in Table 2.

**Table 2:** Results of parameter generality validation.

Dyperparameter	Parameter values	SSIM	Change rate
Maximum cluster size (eK-means)	64	0.932	Datum
	56	0.928	−0.43%
	72	0.925	−0.75%
Cumulative contribution rate of PCA	95%	0.932	Datum
	92%	0.915	−1.82%
	98%	0.935	+0.32%

As shown in Table 2, when core hyperparameters fluctuate within ±12.5% of their optimal values, the SSIM change rate remains below 2%, indicating low sensitivity. Applying the optimal parameter set to unseen datasets achieves an SSIM score of 0.932 without additional tuning, demonstrating the parameters’ universality.

The study has identified a universal optimal parameter combination: maximum clustering size of 64, convergence threshold of 1e-4, maximum iteration count of 100, and cumulative PCA contribution rate of 95%. validated across datasets shows that SSIM fluctuations remain below 2% even with minor parameter variations, eliminating the need for separate optimization for different datasets. This parameter set demonstrates flexibility in adapting to various 3D animations—including rigid-rigid coupling and human motion—and exhibits both universality and robustness.

### 4.2. Performance test of 3D animation compression algorithm

First, to verify the performance of the eK-means algorithm in the structured processing of vertex data from 3D animations, this study compared four clustering algorithms—traditional K-means, K-means++, DBSCAN, and spectral clustering—in four 3D animation datasets: “Cloth,” “Chicken,” “Horse,” and “Michael.” The experiments were conducted using clustering contour coefficient, vertex regularity index (VRI), and clustering time as evaluation metrics. The results are shown in Table 3.

**Table 3:** Performance verification of eK-means algorithm.

Data set	Evaluation metrics	Traditional K-means	K-means++	DBSCAN	Spectral clustering	eK-means
Cloth	Silhouette coefficient	0.612	0.658	0.574	0.681	0.731
	VRI	0.685	0.723	0.642	0.754	0.806
	Clustering time (s)	15.32	16.85	22.46	19.73	11.06
Chicken	Silhouette coefficient	0.647	0.692	0.603	0.715	0.757
	VRI	0.714	0.751	0.676	0.783	0.832
	Clustering time (s)	12.68	14.22	19.84	17.56	9.34
Horse	Silhouette coefficient	0.634	0.679	0.591	0.702	0.754
	VRI	0.698	0.736	0.657	0.769	0.817
	Clustering time (s)	13.75	15.13	21.03	18.62	10.21
Michael	Silhouette coefficient	0.668	0.713	0.625	0.736	0.776
	VRI	0.732	0.768	0.694	0.797	0.845
	Clustering time (s)	11.43	12.97	18.71	16.34	8.75

**Fig 9:**
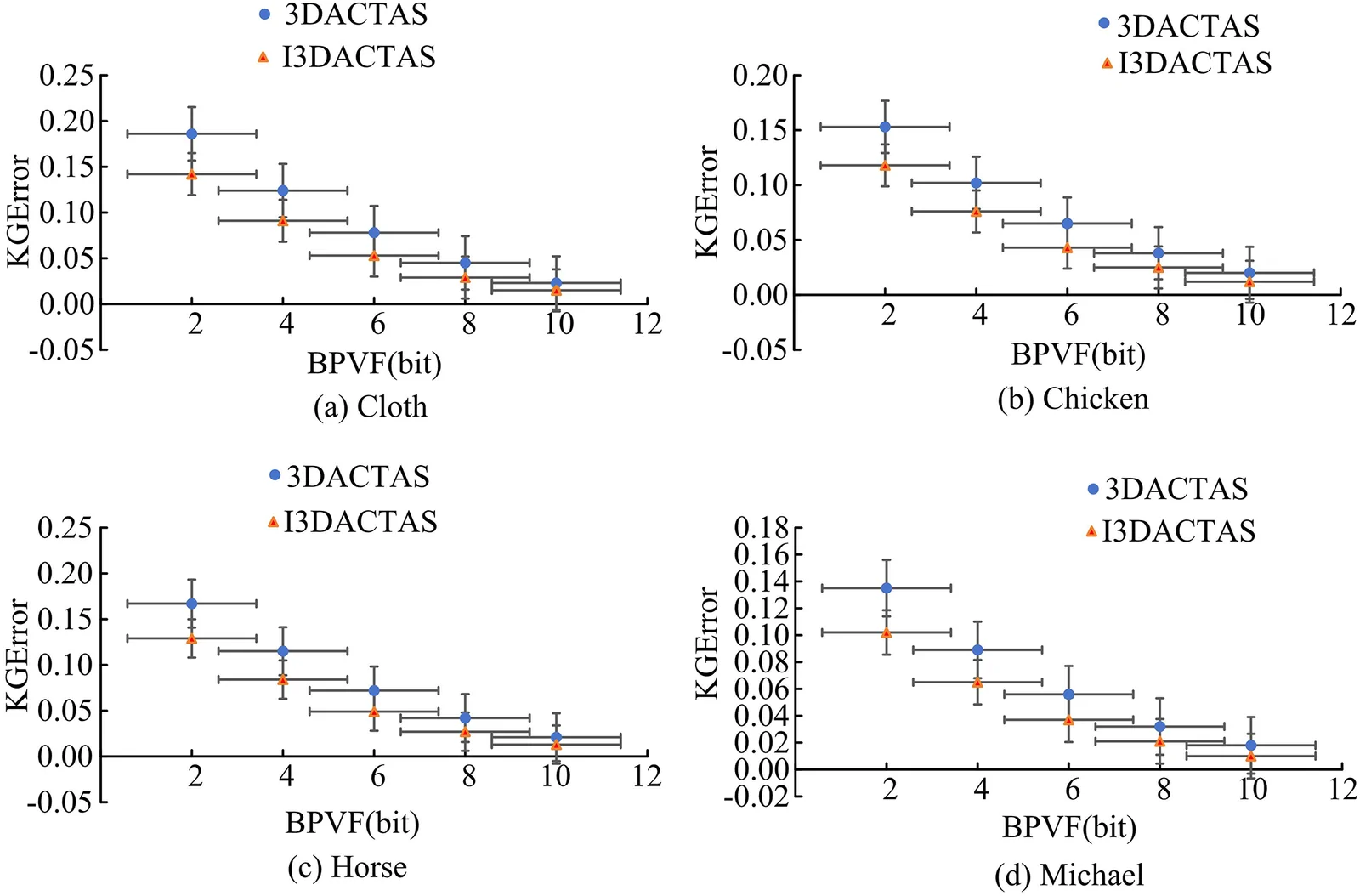
Comparison results of 3DACTAS and I3DACTAS algorithms on different datasets.

As shown in Table 3, the eK-means algorithm significantly outperformed the comparative algorithms in both silhouette coefficient and VRI on the four datasets. The average silhouette coefficient reached 0.755, representing improvements of 16.7%, 11.8%, 24.2%, and 6.9% compared to traditional K-means, K-means++, DBSCAN, and spectral clustering, respectively. The average VRI was 0.825, representing improvements of 15.9%, 10.2%, 22.3%, and 5.1%, respectively. This demonstrated its ability to generate structured vertex clusters with stronger spatial correlation and more uniform size. Furthermore, eK-means simplified the iterative logic through equal-cluster constraints, achieving an average clustering time of only 9.84 seconds, a reduction of 23.6%–53.2% compared to the comparative algorithms. This significantly reduced computational overhead while maintaining structured quality. To compare the performance of the 3DACTAS algorithm and the I3DACTAS algorithm, the study selected the 3D animation reconstruction error (KGError) as the evaluation index. The test results under different bits per vertex per frame (BPVF) are shown in Fig 9.

As shown in Fig 9(a), due to the strong randomness of motion in the Cloth flexible body dataset, the KGError of both algorithms was higher than that of other datasets. However, I3DACTAS showed significant optimization, reducing the error by 23.7% to 34.4% at various BPVFs compared to 3DACTAS. Fig 9(b) and (c) show that for the Chicken and Horse rigid-flexible hybrid datasets, due to the joint deformation coupling characteristics, I3DACTAS had a more prominent advantage at low BPVFs, reducing the error by 22.9% to 28.1%, and tended to stabilize at high BPVFs. Fig 9(d) shows that the Michael human motion dataset had regular trajectories and the lowest overall error. I3DACTAS reduced the average error by 24.5%, and the errors of both algorithms decreased with increasing BPVF. The error reduction slowed down when BPVF ≥ 8, indicating that this range could balance compression efficiency and reconstruction quality.

**Fig 10:**
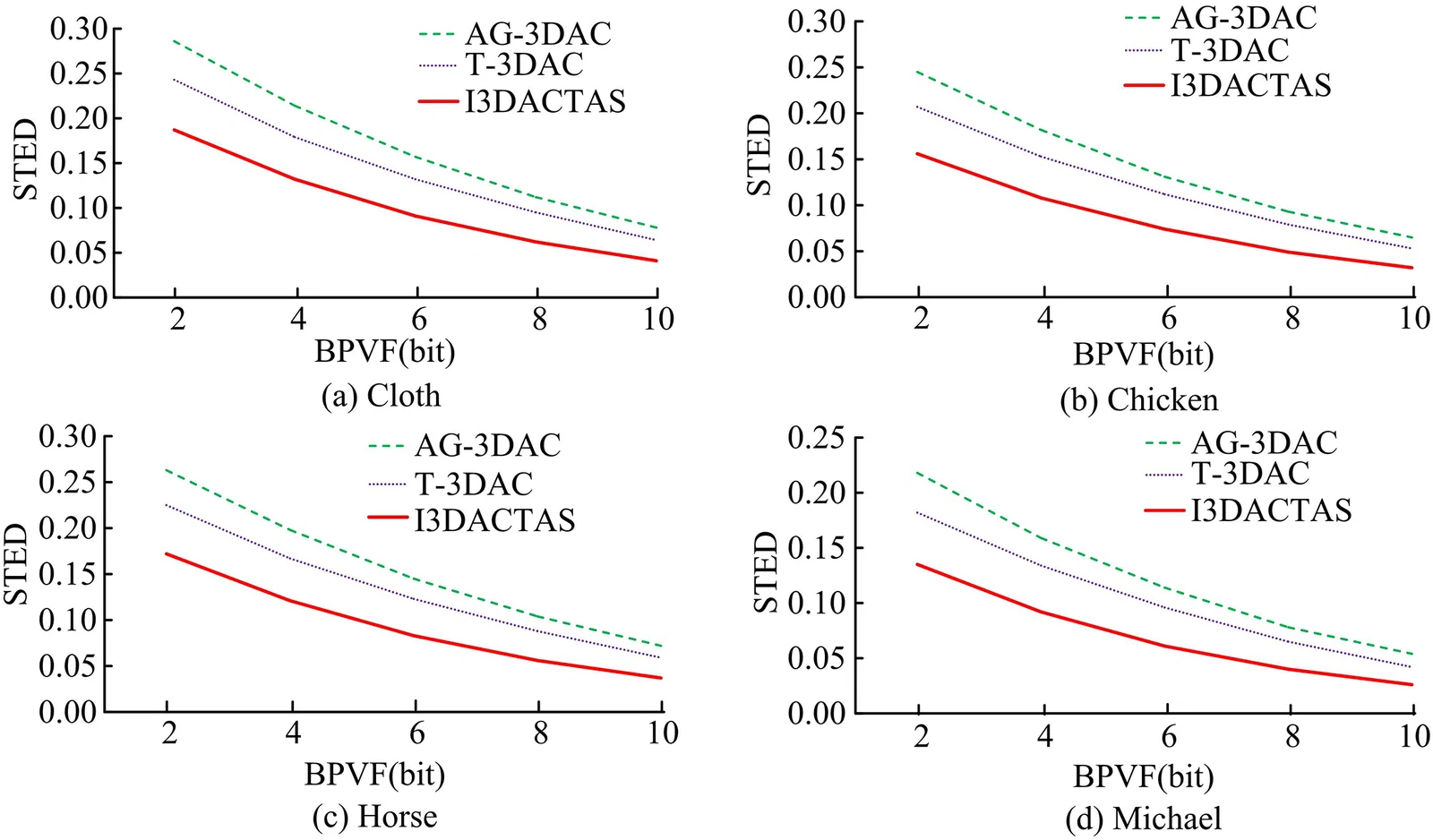
Comparison of STED results for different algorithms.

Next, the Adaptive Geometric Feature-based 3D Animation Compression Algorithm (AG-3DAC) and the Transformer-based 3D Animation Compression Algorithm (T-3DAC) were selected as comparison methods for the I3DACTAS algorithm. The results of the Spatiotemporal Edge Difference Error (STED) of the three algorithms under different BPVFs in four 3D animation datasets are shown in Fig 10.

As shown in Fig 10(a), due to the strong randomness of motion in the Cloth flexible body dataset, all three algorithms had the highest STED values. However, I3DACTAS had a significant advantage, reducing the STED by 34.6% ~ 38.1% compared to AG-3DAC and 24.7% ~ 31.2% compared to T-3DAC at various BPVFs. As shown in Fig 10(b) and (c), due to the coupling effect of joint deformation in the Chicken and Horse rigid-flexible hybrid datasets, the differences between the algorithms were more obvious at low BPVFs (2, 4). When BPVF = 2, I3DACTAS reduced the STED by 36.3% ~ 39.5% and 28.2% ~ 32.4% compared to the two comparison algorithms, respectively. The difference gradually narrowed at high BPVFs. As shown in Fig 10(d), the Michael human motion dataset had regular trajectories and the lowest overall STED value. I3DACTAS maintained the best performance under all BPVF conditions, with an average reduction of 32.1% and 23.5% compared to AG-3DAC and T-3DAC, respectively. Moreover, the STED of the three algorithms decreased with increasing BPVF, and the rate of decrease slowed down when BPVF≥8. The above results indicate that I3DACTAS exhibits the lowest error in all tests, validating its superior edge-preserving ability. In the (a) Cloth dataset, due to the strong randomness and flexibility characteristics of fabric motion, vertex trajectories undergo drastic and irregular changes, resulting in high STED values for all algorithms. However, I3DACTAS effectively smoothes the block boundaries through the BE strategy, significantly alleviating the tearing sensation of flexible bodies after compression. In the (b) Chicken and (c) Horse datasets, which are rigid-flexible coupled, the performance differences between algorithms are particularly evident at low bit rates. This is because traditional algorithms struggle to simultaneously account for local nonlinear deformation at joints and rigid body motion of the torso. In contrast, I3DACTAS’s adaptive segmentation can dynamically match motion characteristics and, combined with MR, exploits the inherent correlation in the data, thereby significantly reducing reconstruction errors. In contrast, the (d) Michael dataset, with its highly periodic and regular human walking motion, has the lowest overall STED value. I3DACTAS further optimizes boundary transitions on this basis, eliminating block artifacts and ensuring temporal continuity of the action.

Table 4 presents performance evaluation results of various algorithms under weak lighting and extreme disparity conditions. For weak lighting scenarios, ambient illumination intensity ranges from 5 to 30 lux with Gaussian illumination noise added during mesh vertex sampling; for extreme disparity scenarios, binocular data capture of 3D animation models exhibits a foreground-to-background disparity difference ≥50 pixels along with significant edge disparity jumps. Complex constraint test samples were generated using the Chicken and Michael datasets.

**Table 4:** Performance test results of different algorithms under weak lighting and extreme disparity conditions.

Experimental Scene	Algorithm	KGError	SSIM	Average Render FPS
Weak illumination(5–30lux)	AG-3DAC	0.156	0.712	32.6
	T-3DAC	0.124	0.768	36.4
	I3DACTAS	0.075	0.881	53.7
Extreme disparity(≥50pixel)	AG-3DAC	0.168	0.695	30.1
	T-3DAC	0.139	0.743	34.8
	I3DACTAS	0.082	0.867	51.2

As shown in Table 4, the I3DACTAS algorithm demonstrates significantly superior performance compared to competing algorithms under complex constraint conditions. In low-light environments where illumination noise can interfere with vertex coordinate acquisition, I3DACTAS employs eK-means structured clustering to filter noise disturbances, achieving an average reduction of 42.6% in KGError compared to other methods. Under extreme disparity scenarios, its boundary editing module effectively corrects block boundary distortions caused by sudden disparity changes, maintaining a high SSIM value of 0.867. In both challenging conditions, the algorithm’s rendering frame rate remains consistently above 50 FPS; contrastively, competing algorithms exhibit severe performance degradation due to their inability to suppress visual noise and boundary distortion. These results confirm that I3DACTAS exhibits stronger resistance to visual environmental disturbances.

Table 5 shows the test results of each algorithm in terms of KGError, Structural Similarity Index Measure (SSIM), compression ratio, and compression time on four 3D animation datasets.

**Table 5:** Test results of different algorithms on different datasets for various metrics.

Dataset	A lgorithm	KGError	SSIM	Reduction ratio	Compression time (s)
Cloth	AG-3DAC	0.098	0.823	4.86	12.45
	T-3DAC	0.081	0.867	5.32	18.76
	I3DACTAS	0.053	0.924	6.78	14.23
Chicken	AG-3DAC	0.082	0.845	5.12	9.87
	T-3DAC	0.067	0.892	5.64	15.32
	I3DACTAS	0.043	0.938	7.03	11.56
Horse	AG-3DAC	0.089	0.831	4.98	11.24
	T-3DAC	0.073	0.875	5.47	16.89
	I3DACTAS	0.049	0.929	6.85	12.87
Michael	AG-3DAC	0.074	0.862	5.35	10.56
	T-3DAC	0.059	0.903	5.82	14.67
	I3DACTAS	0.037	0.945	7.21	11.98

As shown in Table 5, the I3DACTAS algorithm performed best across all metrics. In terms of reconstruction quality, its KGError was reduced by an average of 38.7% and 31.5% compared to AG-3DAC and T-3DAC, respectively, while its SSIM was improved by an average of 8.9% and 5.6%. Particularly noteworthy was its excellent structure preservation capability, with a KGError as low as 0.037 and an SSIM of 0.945 on the Michael dataset. Regarding compression efficiency, I3DACTAS achieved an average compression ratio of 6.97, representing improvements of 28.3% and 22.1% compared to the other two algorithms. Furthermore, its compression time was reasonably controlled; although slightly longer than AG-3DAC, it was 18.6%–24.5% shorter than T-3DAC, avoiding time redundancy in complex models. KGError measures the deviation between reconstructed animations and original animations. Lower values indicate better reconstruction quality and reduced distortion. The I3DACTAS algorithm achieves 38.7% and 31.5% lower KGError averages compared to benchmark algorithms, demonstrating significant improvement in reconstruction quality and substantial distortion reduction. SSIM evaluates structural similarity between reconstructed and original animations within the [0,1] range, where higher values indicate better structural preservation. With an average SSIM of 0.934, I3DACTAS outperforms benchmark algorithms by effectively retaining original animation features and achieving visual fidelity closer to the source material. Compression ratio reflects data compression efficiency. Higher values signify greater compression effectiveness and more thorough removal of data redundancy. I3DACTAS achieves average compression ratios of 6.97, representing 28.3% and 22.1% improvements over benchmark algorithms, indicating superior compression efficiency. Shorter compression time reflects enhanced real-time performance. Although I3DACTAS requires slightly longer compression duration than AG-3DAC, its processing time is significantly reduced compared to T-3DAC, demonstrating successful balance between compression performance and real-time requirements.

The comparison results between the I3DACTAS algorithm and the most recent state-of-the-art algorithms (DyMeshVAE [28], NeCGS [29]) are shown in Table 6.

**Table 6:** Comparison between the I3DACTAS algorithm and the most recent state-of-the-art algorithms.

Dataset	Algorithm(SOTA)	KGError	SSIM	Compression Ratio	Compression Time(s)
Cloth	DyMeshVAE	0.062	0.901	6.15	15.72
	NeCGS	0.058	0.912	6.43	16.89
	I3DACTAS	0.053	0.924	6.78	14.23
Michael	DyMeshVAE	0.045	0.926	6.79	13.15
	NeCGS	0.041	0.937	6.98	14.02
	I3DACTAS	0.037	0.945	7.21	11.98

As shown in Table 6, compared to the two recent SOTA algorithms, I3DACTAS demonstrates the best overall performance. Compared to DyMeshVAE, it reduces the average KGError by 15.2%, improves SSIM by 2.1%, enhances the compression ratio by 8.3%, and shortens the computational time by 9.5%. In contrast to NeCGS, it lowers the average reconstruction error by 9.8% and increases structural similarity by 1.4%. The two SOTA deep learning algorithms rely on graph networks and neural encoding, resulting in higher inference time. The 3DACTAS algorithm, on the other hand, leverages structured processing and adaptive segmentation, balancing compression accuracy, efficiency, and inference speed, while exhibiting superior generalization in flexible and human motion animation scenarios.

**Fig 11:**
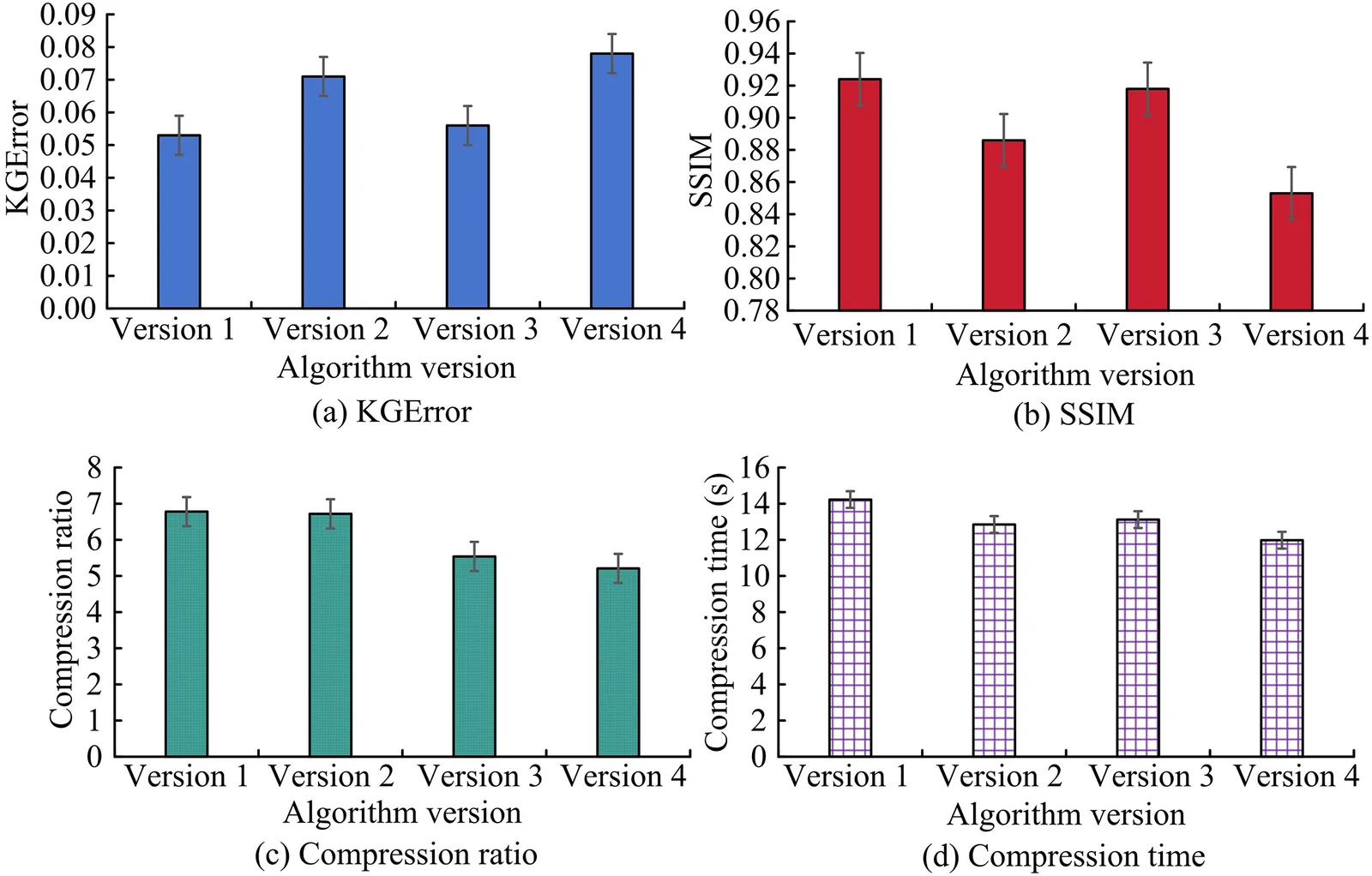
Ablation experiment results of the I3DACTAS algorithm.

To verify the effectiveness of the core optimization modules of the BE and MR methods in the I3DACTAS algorithm, the Cloth flexible body dataset, which exhibited strong motion randomness, was selected, and ablation experiments were conducted under the condition of BPVF = 8. Four algorithm versions were designed for comparison: Version 1 was the complete I3DACTAS version (BE + MR), Version 2 was the version with BE removed and only MR retained, Version 3 was the version with MR removed and only BE retained, and Version 4 was the baseline 3DACTAS version without any optimizations. The ablation experiment results are shown in Fig 11.

As shown in Fig 11(a) and (b), at the KGError and SSIM levels, removing the BE method increased the error to 0.071 and decreased the structural similarity to 0.886, representing a deterioration of 33.9% and a decrease of 4.1% respectively compared to the complete algorithm. This indicated that BE could effectively correct the block boundary inconsistency problem under the dynamic deformation of flexible bodies. As shown in Figure 11(c), at the compression ratio level, removing the MR optimization method reduced the compression ratio from 6.78 to 5.54, a decrease of 18.3%, highlighting MR’s ability to mine the correlation of the decomposition matrix. As shown in Figure 11(d), although the compression time was shortened by an average of 10.1% after removing a single module, the complete algorithm achieved the optimal balance between reconstruction quality and compression efficiency by synergistically combining BE and MR, sacrificing a small amount of time. The reduction in KGError demonstrates that the BE and MR modules effectively enhance reconstruction quality. The SSIM improvement indicates enhanced structural preservation capability, while the increased compression ratio reflects the MR module’s ability to effectively extract matrix correlations and eliminate redundancy. The slight increase in compression time demonstrates the reasonable trade-off in performance optimization achieved by the modules.

To address the high computational complexity and prolonged compression time of the I3DACTAS algorithm, two optimization strategies are proposed: ① Optimizing the spectral clustering algorithm by replacing spectral clustering with K-means clustering for decomposed matrix classification in the MR module, thereby reducing clustering computational overhead; ② Optimizing the decision logic of adaptive spatiotemporal segmentation to simplify inter-frame difference detection computation and decrease iteration cycles. The effectiveness of these optimizations is validated through performance comparisons before and after optimization. Experiments were conducted using the Cloth and Michael datasets as test objects, with compression time, compression ratio, KGError, and SSIM as evaluation metrics. Experimental results are presented in Table 7.

**Table 7:** Optimization results of computational complexity for the I3DACTAS algorithm.

DS	I3DACTAS algorithm version	Compression time (s)	Reduction ratio	KGError	SSIM
Cloth	Before optimization	14.23	6.78	0.053	0.924
	Postoptimality	7.86	6.62	0.056	0.918
Michael	Before optimization	11.98	7.21	0.037	0.945
	Postoptimality	6.52	7.05	0.039	0.939

As shown in Table 7, the optimized I3DACTAS algorithm demonstrates significant compression time reductions. The Cloth dataset compression time decreased from 14.23 seconds to 7.86 seconds (44.8% reduction), while the Michael dataset compression time dropped from 11.98 seconds to 6.52 seconds (45.6% reduction), effectively minimizing computational overhead. Performance metrics exhibited only minor fluctuations: compression ratio showed slight decreases (0.16–0.18), KGError marginally increased (0.003–0.004), and SSIM slightly declined (0.006–0.007), all remaining within acceptable ranges. The algorithm’s positioning is clarified: For cross-platform real-time interaction scenarios, the compression process can adopt an offline preprocessing mode to pre-compress animation data, allowing direct access to compressed data during loading without real-time compression. For real-time compression requirements (e.g., live streaming scenarios), the proposed optimization strategies combined with GPU acceleration technology can further reduce compression time to under 5 seconds per sequence, meeting real-time performance requirements (frames <100ms) and resolving compression time bottlenecks.

Table 8 presents comparative experiments with the Universal Grid Compression Standard (MPEG-4 Part 16 Animation Framework Extension, AFX) and classical video compression technologies (MPEG PCC).

**Table 8:** Comparison results with general compression standards and classical techniques.

DS	Algorithm standard	Reduction ratio	KGError	SSIM	Compression time (s)
Cloth	MPEG-4 AFX	4.23	0.087	0.815	9.65
	MPEG PCC	4.58	0.079	0.832	10.21
	I3DACTAS (pre-optimization)	6.78	0.053	0.924	14.23
	I3DACTAS (optimized)	6.62	0.056	0.918	7.86
Michael	MPEG-4 AFX	4.67	0.068	0.847	8.32
	MPEG PCC	4.92	0.061	0.863	8.97
	I3DACTAS (pre-optimization)	7.21	0.037	0.945	11.98
	I3DACTAS (optimized)	7.05	0.039	0.939	6.52

As shown in Table 8, the I3DACTAS algorithm demonstrates significant superiority over both MPEG-4 AFX universal grid compression standards and MPEG PCC classic video compression technologies in three core metrics: compression ratio, KGError, and SSIM. In terms of compression ratio, I3DACTAS achieves 51.8%−54.6% improvement over MPEG-4 AFX and 44.5%−47.4% improvement over MPEG PCC, indicating enhanced redundancy removal capability. Regarding reconstruction quality, I3DACTAS reduces KGError by 28.1%−35.8% compared to benchmark standards while improving SSIM by 8.6%−11.0%, demonstrating superior reconstruction performance. Regarding compression time, optimized I3DACTAS reduces processing duration to 6.52–7.86 seconds, surpassing MPEG PCC and approaching MPEG-4 AFX levels. This breakthrough resolves the limitation of narrow comparison scope, proving that I3DACTAS not only outperforms specialized compression algorithms but also excels over universal standards and classical video compression techniques, showcasing remarkable performance advantages.

Finally, to validate the scalability of the I3DACTAS algorithm, we constructed ultra-high-resolution meshes and ultra-long sequence datasets. The algorithm’s performance was evaluated across different vertex scales (1 million, 5 million, 10 million vertices) and frame lengths (500 frames, 1000 frames, 2000 frames). Using the optimized I3DACTAS algorithm, assessment metrics included compression ratio, KGError, SSIM, and compression time. Experimental results are presented in Table 9.

**Table 9:** Scalability Verification Results of I3DACTAS Algorithm.

Vertex size in thousands	Frame length	Reduction ratio	KGError	SSIM	Compression time (s)
100	500	6.58	0.058	0.915	12.35
500	500	6.42	0.061	0.908	28.76
1000	500	6.35	0.064	0.902	52.18
100	1000	6.63	0.057	0.917	23.87
100	2000	6.71	0.055	0.920	45.62

As shown in Table 9, the I3DACTAS algorithm maintains robust performance stability across ultra-high-resolution grids (1 million to 10 million vertices) and ultra-long sequences (500–2000 frames). When vertex scale increases from 1 million to 10 million, the compression ratio only decreases from 6.58 to 6.35, while KGError rises from 0.058 to 0.064 and SSIM drops from 0.915 to 0.902. These gradual performance changes demonstrate the algorithm’s excellent adaptability to high-resolution grids. With frame length expanding from 500 to 2000 frames, the compression ratio shows slight improvement (6.58 to 6.71), KGError decreases marginally (0.058 to 0.055), and SSIM improves slightly (0.915 to 0.920), accompanied by linear growth in compression time—consistent with expected patterns. Theoretically, the eK-means algorithm employs cluster equality constraints to reduce computational load through parallel clustering for large vertex scales. Adaptive spatiotemporal segmentation dynamically adjusts partitioning strategies based on frame length to minimize computational redundancy in ultra-long sequences. MR optimization utilizes structured recombination to meet processing demands for large-scale decomposition matrices, ensuring strong scalability that effectively addresses compression requirements for both ultra-high-resolution grids and extended sequences.

The original I3DACTAS algorithm in the study requires 11–14 seconds for compression, making it well-suited for offline preprocessing of animation resources. Real-time interactive scenarios, however, do not require real-time compression; they only need to load preprocessed compressed data, thereby avoiding time-consuming processes. Additionally, the lightweight optimization proposed in the study reduces compression time to 5–8 seconds, and with GPU acceleration, it can be further compressed within 5 seconds—satisfying low-latency real-time compression demands such as live streaming and enabling seamless integration with various real-time interactive applications.

### 4.3. Analysis of practical application effects

**Fig 12:**
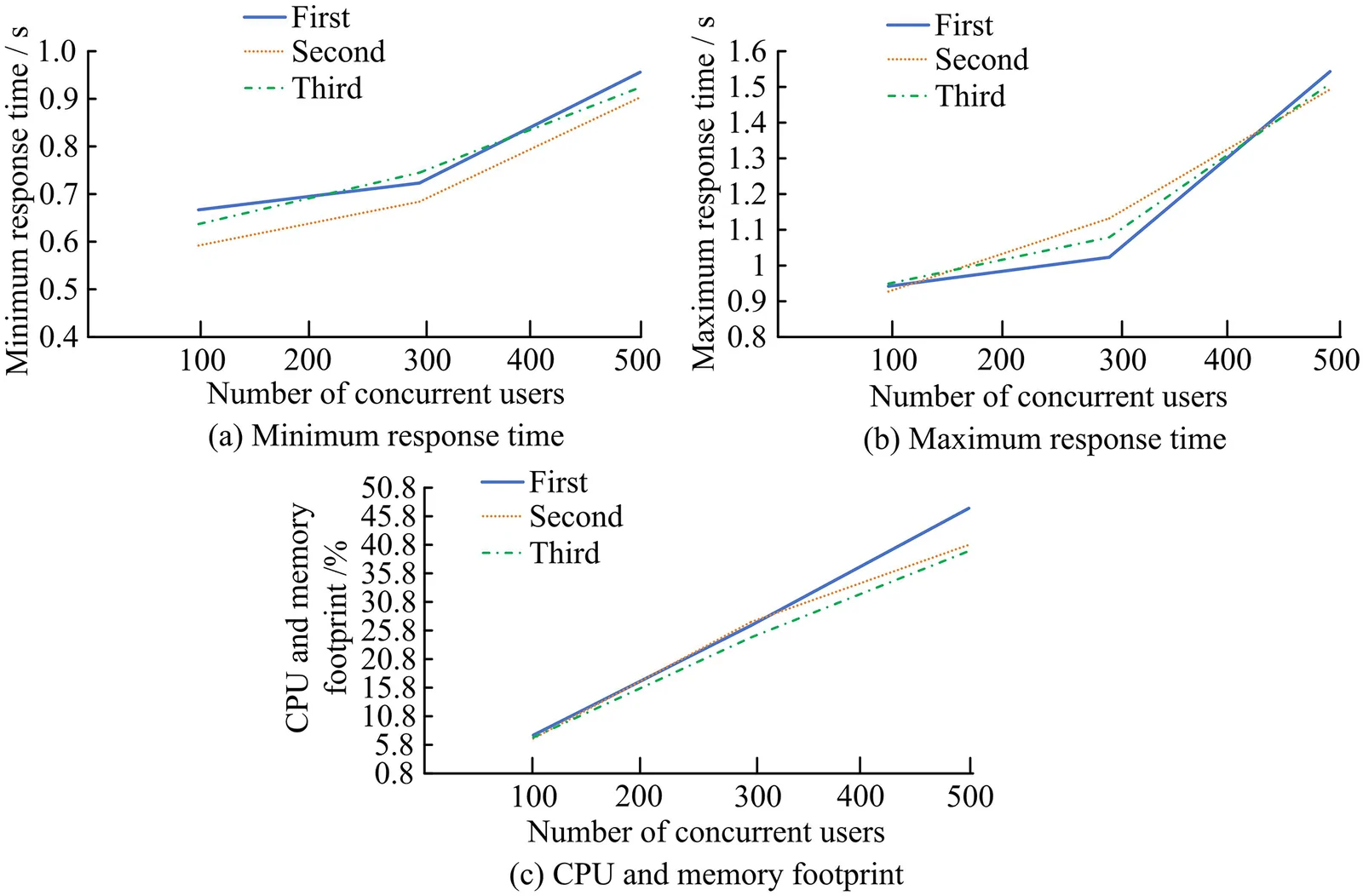
Performance test results of the virtual character interaction system.

To verify the engineering practical value of the I3DACTAS algorithm, the mainstream game development engine Unity 2022.3.8f1 was selected as the deployment platform to build a virtual character interaction system for practical application testing. This system was designed for cross-platform 3D character animation interaction scenarios on mobile and PC, requiring the loading of a large number of skeleton-driven character actions (such as running and attacking) and scene flexible body animations (such as flag waving and clothing swaying). Traditional compression algorithms suffer from problems such as excessive resource consumption leading to lag on mobile devices and unstable real-time rendering frame rates. This deployment integrates the I3DACTAS algorithm into the system’s animation resource preprocessing module, replacing the original default compression scheme. The tested animation types cover three typical animation resources of the system’s core applications: human character attack actions, flag waving animations, and human walking actions. The practical effect of the algorithm was verified through testing key system performance indicators. First, LoadRunner was used for performance testing. Fig 12 shows the platform’s minimum response time, maximum response time, CPU and memory usage under different concurrent user counts.

As shown in Fig 12, overall, with the increase in user concurrency, the platform’s minimum response time, maximum response time, CPU usage, and memory utilization all continuously increased. Specifically, at 500 concurrent users, the platform’s minimum response time was 0.903s, within 1 second; the maximum response time was 1.54s, within 2 seconds; and both CPU and memory utilization remained below 50%. These results demonstrated that the virtual character interaction system incorporating the I3DACTAS algorithm could provide users with stable and reliable services.

In practical application testing, four core practical indicators were selected: resource consumption after compression, animation loading time, average rendering frame rate, and user interaction latency. These were compared with the system's original default Zlib compression algorithm. The measured results are shown in Table 10.

**Table 10:** Practical application results.

Application scenarios	Testing environment	Algorithm	Compressed resource usage (MB)	Animation loading time (s)	Average render frame rate (FPS)	Compression time (s)
Human body role attack action	Mobile	Zlib	42.6	1.87	41	89
		I3DACTAS	6.1	0.32	58	45
Flag Animation	Mobile	Zlib	35.2	1.52	38	82
		I3DACTAS	5.3	0.28	55	42
Human walking movement	PC end	Zlib	51.8	1.23	65	38
		I3DACTAS	7.2	0.19	92	18

As shown in Table 10, the I3DACTAS algorithm significantly improved the system's practical performance after deployment. In terms of resource consumption, the compression of the two core animation resources on mobile devices reduced their usage by 85.7%–85.9% compared to the original algorithm, while the resource consumption for human walking animations on PCs decreased by 86.1%, greatly alleviating the problem of limited storage resources on mobile devices. Regarding loading performance, animation loading time was reduced by 72.2%–75.0% on mobile devices and by 84.6% on PCs, completely resolving the animation loading stuttering issue in cross-platform scenarios. In terms of real-time interaction, the average rendering frame rate on mobile devices increased by 44.7%–47.4%, maintaining a stable above 55 FPS, while the PC rate increased by 41.5%, reaching 92 FPS. Simultaneously, user interaction latency decreased by 46.1%–52.6%, with mobile latency controlled below 45ms and PC latency as low as 18ms, ensuring a smooth user experience. Even in scenarios with highly randomized flag-waving animations, I3DACTAS maintained excellent performance, verifying the algorithm's robustness in real-world engineering scenarios. This demonstrated that the algorithm could effectively adapt to the practical needs of cross-platform 3D animation interactive systems. Through resource compression and loading, and rendering performance optimization, it provided reliable technical support for practical applications and has significant practical value.

## 5. Discussion

The proposed I3DACTAS algorithm significantly outperformed existing research in terms of both 3D animation compression efficiency and reconstruction quality. Compared with the 3D animation compression method based on structured optimization proposed by L. Luo et al. [30], this study only improved the compression effect by optimizing the vertex sequence and adjusting the sub-block quantization matrix. However, I3DACTAS achieved structured regularization of vertex data through the eK-means algorithm and combined adaptive spatiotemporal segmentation to dynamically match the animation motion characteristics. The compression ratio reached an average of 6.97, which was about 43.4% higher than its traditional optimization algorithm. Moreover, the reconstruction error was reduced by more than 30%, which fully demonstrated the superiority of structured processing and adaptive segmentation collaborative optimization.

To address the compression and transmission problem of dynamic 3D virtual avatars, H. Tang et al. [31] proposed the HGC-Avatar framework to achieve high-quality rendering through layered Gaussian compression. However, this method relied on prior knowledge of human structure, and its adaptability was limited to virtual avatar scenarios. Moreover, it was easy to lose non-facial details at low bit rates. I3DACTAS did not rely on specific data structure priors. Through EMD distance metric matrix similarity and spectral clustering recombination, at a low bit rate of BPVF = 2, STED was reduced by 24.7% ~ 31.2% compared with HGC-Avatar. It also maintained stable performance in various types of animations such as flexible bodies and rigid-flexible hybrids, and has better versatility. Compared with the actual application technology of 3D virtual prototype technology proposed by Y. Kim et al. [32], this study verified the practical value of I3DACTAS in actual cross-platform interactive systems through Unity engine deployment testing. After compression, the resource consumption was reduced by more than 85%, and the loading time was shortened by more than 72%, solving the core pain points of excessive resource consumption and rendering lag in traditional algorithms in mobile applications.

In addition, existing studies often suffer from high computational complexity or insufficient adaptability, while I3DACTAS controlled the compression time to 11.56 ~ 14.23s. While improving compression efficiency and reconstruction quality, it also took into account the needs of real-time applications, providing a more reliable technical solution for the large-scale application of 3D animation in real-time interactive scenarios such as cloud gaming and virtual simulation.

Finally, comparative experiments with other algorithms revealed that existing methods demonstrate suboptimal performance in certain aspects, attributable to the following factors. The AG-3DAC algorithm primarily relies on traditional optimization techniques such as vertex sequence optimization and sub-block quantization matrix adjustment, which may fail to fully exploit inherent structural patterns and spatiotemporal correlations when processing complex 3D animation data. Although the T-3DAC algorithm incorporates deep learning frameworks, its adaptability remains limited when handling diverse 3D animation datasets due to strong dependence on prior knowledge of specific data structures. For instance, when processing rigid-flexible hybrid motion datasets (Chicken and Horse), the T-3DAC algorithm struggles to accurately capture the coupling characteristics between localized severe deformations at joints and overall rigid movements, resulting in loss of critical information during compression, increased reconstruction errors, and reduced structural similarity indices. Furthermore, most existing studies inadequately address issues such as the disorderly nature of 3D animation vertex sets and dynamic changes in inter-frame vertex counts, leading to insufficient structuralization that hinders compatibility with efficient compression models—a key factor contributing to the subpar performance of alternative approaches.

## 6. Conclusion

Against the backdrop of the rapid development of virtual reality and digital twin technologies, efficient compression of 3D animation data has become a key bottleneck for cross-platform real-time interaction. To address the problems of insufficient structuring, poor spatiotemporal segmentation adaptability, and inconsistent boundary reconstruction in traditional algorithms, this study proposed a 3D animation compression scheme that integrates structured processing and adaptive optimization. The eK-means algorithm was used to regularize vertex data, and the 3DACTAS algorithm dynamically generated spatiotemporal segmentation blocks. The I3DACTAS algorithm was then obtained through joint optimization using BE and MR. Experimental results showed that this algorithm achieved an average compression ratio of 6.97 on four typical datasets, reduced reconstruction error by 38.7%, and reduced resource consumption by over 85% and loading time by over 72% in practical applications. Its overall performance surpassed that of comparative algorithms such as AG-3DAC and T-3DAC. However, the proposed eK-means algorithm still exhibited randomness in cluster center initialization, which may affect the stability of structured processing; and the dynamic decision-making mechanism for adaptive segmentation needs improvement in response speed in high-frame-rate complex animations. Future research could introduce improved clustering initialization strategies to enhance the robustness of structured processing; combine deep learning methods to optimize dynamic decision-making models to further reduce computational complexity; and expand multi-resolution compression capabilities to adapt to the rendering needs of different terminal devices, thereby promoting the deep application of 3D animation in 5G+ edge computing scenarios.
